# How and Why Are Cancers Acidic? Carbonic Anhydrase IX and the Homeostatic Control of Tumour Extracellular pH

**DOI:** 10.3390/cancers12061616

**Published:** 2020-06-18

**Authors:** Shen-Han Lee, John R. Griffiths

**Affiliations:** 1 Department of Otorhinolaryngology, Hospital Sultanah Bahiyah, Jalan Langgar, Alor Setar 05460, Kedah, Malaysia; 2 Cancer Research UK Cambridge Institute, University of Cambridge, Li Ka Shing Centre, Robinson Way, Cambridge CB2 0RE, UK; john.griffiths@cruk.cam.ac.uk

**Keywords:** cancer microenvironment, tumour pH, carbonic anhydrase IX, cancer metabolism, pH-stat, pH measurement in vivo, magnetic resonance spectroscopy, models of tumour pH regulation

## Abstract

The acidic tumour microenvironment is now recognized as a tumour phenotype that drives cancer somatic evolution and disease progression, causing cancer cells to become more invasive and to metastasise. This property of solid tumours reflects a complex interplay between cellular carbon metabolism and acid removal that is mediated by cell membrane carbonic anhydrases and various transport proteins, interstitial fluid buffering, and abnormal tumour-associated vessels. In the past two decades, a convergence of advances in the experimental and mathematical modelling of human cancers, as well as non-invasive pH-imaging techniques, has yielded new insights into the physiological mechanisms that govern tumour extracellular pH (pH_e_). In this review, we examine the mechanisms by which solid tumours maintain a low pH_e_, with a focus on carbonic anhydrase IX (CAIX), a cancer-associated cell surface enzyme. We also review the accumulating evidence that suggest a role for CAIX as a biological pH-stat by which solid tumours stabilize their pH_e_. Finally, we highlight the prospects for the clinical translation of CAIX-targeted therapies in oncology.

## 1. Introduction

The pH of extracellular fluid (pH_e_) in healthy tissues is tightly regulated between 7.35 and 7.45, in order to sustain normal physiology and cellular metabolism. In contrast, the pH_e_ of cancers is acidic, between 6.3 and 7.0, reflecting the dysregulation of the acid-base homeostatic mechanisms that operate within solid tumours.

Almost a century ago, Otto Warburg published his seminal observation, termed aerobic glycolysis, in which tumour slices metabolized glucose into lactic acid instead of CO_2_, even in the presence of oxygen [1]. This observation, now termed ‘The Warburg Effect’ gave rise to the erroneous assumption that lactic acid produced by cancer cells would tend to acidify their cytoplasm and thus lower their intracellular (pH_i_) [2,3]. Microelectrode studies on solid tumours appeared to support the assertion that tumours were acidic, and for half a century it was believed that both their pH_i_ and their pH_e_ were acidic. That dogma was overturned by the first magnetic resonance spectroscopy (MRS) studies on cancer in the early 1980s [4,5,6], which demonstrated that the pH_i_ of solid tumours, both in animal models and in patients, was usually on the alkaline side of neutrality, much like that of normal cells. Subsequent MRS studies using extracellular pH probes, such as 3-aminopropylphosphonate (3-APP) [7] and the imidazole probes 2-imidazole-1-yl-3-ethoxycarbonyl propionic acid (IEPA) [8] and (±)2-(imidazol-1-yl) succinic acid (ISUCA) [9], firmly established the conclusion that it was the extracellular, interstitial space of tumours that was acidic, with typical pH_e_ values of 6.3–7.0.

In the last two decades, the acidic tumour microenvironment has become recognized as a feature of the tumour phenotype that drives the progression of cancer. It modulates several steps along the spectrum from preneoplasia to neoplasia, and then along the invasion–metastasis cascade, thus conferring an evolutionary advantage on solid tumours. Several reviews have discussed aspects of this process [10,11,12]. In parallel, advances in imaging technology, biophysics and chemistry have converged with the progress in experimental modelling of human cancers to yield novel insights into the mechanisms that cause and regulate the acidic tumour pH_e_; the enzyme carbonic anhydrase IX (CAIX) has been found to play a key role in these mechanisms. From a clinical perspective, the acidic tumour pH_e_ is also an emerging diagnostic and therapeutic target. While current targeted therapies in oncology mostly focus on precise molecular targets such as receptor tyrosine kinases [13,14], microRNAs [15,16], or the ubiquitin–proteasome pathway [17,18], the acidic tumour pH_e_ is an attractive target because it is a common phenotype that is found across a wide spectrum of cancers. In this review, we will discuss the common imaging techniques and their applications in various experimental models of cancer. We will also highlight the experimental findings that inform how CAIX regulates tumour pH_e_, and discuss the implications of these findings concerning the pathophysiology of disease progression.

## 2. Methods for Measuring Tumour pH

Techniques for measuring pH in living systems are central to the study of tumour pH_e_. They have been extensively reviewed elsewhere [19,20]; however, we will briefly introduce some of the major techniques to facilitate our subsequent discussion concerning their applications in cancer models.

The first measurements of tissue pH in vivo used microelectrodes, which measure the potential differences of solutions with differing proton concentrations across a thin glass membrane. The electrodes used in living tissues are much larger than cells, so they mainly measure pH_e_. The principle disadvantage of microelectrode pH measurement is that it is invasive and causes damage to cells.

Today, many non-invasive imaging modalities have been developed to measure tissue pH, including magnetic resonance, fluorescence imaging, positron emission tomography (PET) and single-photon emission computerized tomography (SPECT). They rely on a variety of mechanisms to generate image contrast, and have a wide range of spatial resolution. While these techniques have been useful in the preclinical setting, none of them have entered routine medical use, although efforts are underway to translate some of them into the clinic. Table 1 summarizes the range of non-invasive or minimally invasive techniques available for measuring tissue pH_e_.

## 3. Experimental Cancer Models for the Study of Tumour pH Regulation

To gain insight into tumour pH_e_ regulation in vivo, imaging techniques have to be deployed on cancer models ranging from traditional monolayer cell cultures to mouse models and human patients. The measurement of tumour pH_e_ becomes more challenging as the complexity and physiologic relevance of the model system increase.

Two-dimensional (2D) monolayer cell cultures have traditionally been the workhorse of cancer research, and most early studies on cancer pH regulation were done in this system. However, the extracellular environment within the monolayer cultures does not reflect the tumour microenvironment in vivo, not least because H^+^, CO_2_ and HCO_3_^−^ can freely diffuse between the cells and the relatively huge volume of culture medium [42,43]. In contrast, the cancer cells in solid tumours co-exist in a disorganized tissue architecture with stromal fibroblast cells and various immune cells, all of which derive their blood supply from an abnormal tumour-associated vasculature [44]. The restricted diffusive capacity of H^+^, CO_2_ and HCO_3_^−^ in the extracellular space of solid tumours [45] causes spatiotemporal heterogeneity of oxygenation and cellular metabolism, resulting in gradients of pH_e_ that will not occur in 2D cell cultures.

In vitro 3D cell spheroid systems simulate some aspects of the spatial architecture and cell-to-cell contact found in solid tumours in vivo and provide a tractable platform to study the role of several cell physiological processes in regulating the tumour microenvironmental pH [43]. Spheroids are cell cultures in which cells are grown in clusters or aggregates, typically without the addition of the extracellular matrix or growth factors to the culture medium, or by growing cell clusters in low-attachment culture plates. Optical imaging by pH-sensitive fluorescent dyes can be used to measure spatial gradients of pH_i_ and pH_e_ in these spheroid models.

More recently, the application of organoid technology from the field of stem cell research has led to the development of cancer organoids—microscopic self-organizing, 3D structures that recapitulate many of the structural and functional aspects of tumours in vivo [46]. The cancer cells are embedded in a 3D matrix such as a collagen or basement membrane, sometimes with the addition of growth factors that promote growth as a 3D structure. Such organoids can also be co-cultured with fibroblasts and immune cells, thus recreating something of the cellular heterogeneity found within solid tumours. These organoids are increasingly used in the study of tumour metabolism and pH regulation, and they present an improvement over tumour spheroids in modelling in vivo tumour physiology [47,48].

However, tumour pH regulation cannot be fully modelled in culture, even by using tumour spheroids and organoids. Cancer cells in solid tumours are genetically heterogeneous, and they co-exist with non-transformed stromal and immune cells within an unknown nutrient and O_2_ milieu, with further differences due to tumour stage and exposure to therapy. While in situ tumours within patients remain the gold standard for studying tumour pH_e_, animal models allow studies of gene and protein function by analysing the phenotypes that arise after silencing or overexpressing particular genes. Animal models for studying cancer biology differ in their complexity and their degree of fidelity to human cancers [49]. They include genetically engineered mouse models (GEMMs) that develop tumours in situ, as well as allograft models and xenograft models derived from human tumours (patient-derived xenografts) or cancer cell lines. These in vivo models come closer to the physiological complexity of human cancers than cell culture preparations, and they also allow interactions between the tumour and normal host tissues. Nonetheless, the interpretation of the data from mouse models must have the caveat that factors such as diet, strain, sex, age, husbandry and environmental stressors can affect metabolism of the host animal, and thus the metabolism of the tumour [50,51]. Moreover, pH regulation studies in such model systems are restricted by the currently available imaging techniques, which have limited sensitivity, reproducibility and spatiotemporal resolution, and face challenges with the targeting and accessibility of pH probes.

## 4. Why Is Tumour pH_e_ Acidic?

For many years, it was believed that the increased lactate metabolic flux and extrusion of this acidic metabolite from cancer cells explained tumour extracellular acidification. This simple model assumed that lactic acid exported from cancer cells into the interstitial fluid accumulates because it cannot be transported away rapidly enough to the blood vasculature, and that acidification ensues because of the poor buffering capacity of the extracellular fluid.

Early evidence to challenge this concept came from a series of studies by Tannock and colleagues in the 1990s, which found that tumours derived from glycolysis-deficient Chinese Hamster fibroblasts, that lacked both the phosphoglucose isomerase enzyme and a number of glucose transporters, surprisingly had an acidic pH_e_ in vivo, even though negligible amounts of lactic acid were formed and these cells were not able to acidify the culture media in vitro [52]. This finding was replicated using Chinese Hamster ovary cells lacking lactate dehydrogenase, which also showed minimal lactic acid production in vitro but were nonetheless capable of producing tumours with acidic pH_e_ when transplanted in vivo [53]. One possible explanation for this unexpected result would be that these mutant cells produced large amounts of CO_2_ by oxidative metabolism. In fact, earlier studies by Gullino et al., sampling interstitial fluid from solid tumours grown in ‘Gullino’ PTFE chambers, showed that the tumour interstitial fluid of numerous tumour types contained high levels of CO_2_ [54].

Numerous studies on cancer metabolism over the past decade have revealed that cancer cells exhibit aerobic glycolysis due to the activation of oncogenes, the loss of tumour suppressor genes, and the upregulation of the PI3K pathway, and that one advantage of these high glycolytic rates is the availability of precursors for anabolic pathways, that are needed for the biosynthesis of cellular building blocks during proliferation [55,56]. While this Warburg effect has been observed in many human cancers (and is indeed the basis by which FDG-PET scans operate), studies have demonstrated that human cancers show distinct metabolic heterogeneity [57] and produce most of their metabolic energy through glucose oxidation via the tricarboxylic acid cycle, resulting in the formation of CO_2_ [58,59,60].

By sampling the blood entering and exiting solid tumours in vivo, it is possible to calculate their outputs of lactic acid and of CO_2_. Such calculations have been performed on both animal models and tumours in patients, and they have found that the tumour CO_2_ output is comparable to or greater than that of lactate. Kallinowksi et al. studied a tumour xenograft model in which human tumour xenografts were grown in nude rats, on pedicles containing the epigastric artery and vein, and were prevented from acquiring other vasculature by polyethylene sheaths [61,62]. This defined blood supply allowed measurement of the blood flow and assay of metabolite concentrations from the artery supplying and the veins draining the tumour. Holm et al. performed similar balance studies on human colon carcinomas during bowel resection surgery, by cannulating the mesenteric vessels supplying these tumours and measuring the metabolites in blood from the mesenteric artery and vein [63]. Calculations of the production of CO_2_ from the substrate balance data reported by Kallinowski et al. found that human xenografts in nude rats produced 588–850 nmol/g/min CO_2_ vs. 527 nmol/g/min lactate, and similar calculations from the data in Holm et al. found that colonic tumours in human patients produced five times more CO_2_ than lactate, 1296 nmol/g/min CO_2_ vs. 220 nmol/g/min lactate [64]. These balance studies suggested that solid tumours in vivo synthesised most of their ATP by oxidative metabolism (because the complete oxidation of glucose to CO_2_ phosphorylates 16 times the amount of ATP obtained from glycolysis to lactate [65]), and they did not support the dogma that cancer cells rely on aerobic glycolysis for their energetic requirements or that tumour extracellular acidification is entirely due to lactate extrusion.

However, although these in vivo studies suggest that CO_2_ will be a significant source of metabolic acid production in cancer cells, the higher pK of carbonic acid (pKa 6.35) compared to that of lactic acid (pKa 3.86) means that, on a mole for mole basis, CO_2_ would have a much smaller acidifying effect than lactic acid. This suggests that in order for CO_2_ to be a significant source of acidity in the tumour extracellular space, there must be an additional mechanism in play, such as one to accelerate the hydration of CO_2_ to form H^+^. In the next section of this review, we explore how CAIX provides such an additional mechanism and thus maximises the locally acidifying effect of CO_2_ production.

## 5. Carbonic Anhydrase IX and Its Role in Cancer Biology

The role of the enzyme CAIX is central to understanding how CO_2_ production leads to the acidification of the tumour interstitial space. CAIX belongs to a family of zinc metalloprotein enzymes that catalyse the reversible interconversion of CO_2_ to form HCO_3_^−^ and H^+^ [66]. In mammals, there are 16 isoforms of carbonic anhydrases, which differ in their activity and cellular localization [67]. Cancer cells primarily express the plasma membrane-associated carbonic anhydrases, CAIX and CAXII [68,69,70], as well as intracellular carbonic anhydrases such as CAI [71,72] and CAII [73,74,75]. CAIX has been extensively studied as a potential therapeutic target in oncology, owing to its specificity to cancer cells—it has a very restricted expression in the normal stomach and gut epithelial cells [76] but is strongly upregulated in many different types of tumour tissue [77]. Several preclinical studies have suggested that the inhibition of the CAIX function delays the growth of tumours [78,79,80] and reduces invasion and metastasis under experimental conditions [79].

CAIX is a protein that is tethered to the cell surface membrane. It is comprised of an extracellularly facing catalytic domain, an N-terminal proteoglycan-like (PG) domain, a single transmembrane domain and a short intracellular C-terminal tail [70]. The N-terminal PG domain is unique to CAIX within the CA family [81] and has been shown to be important in the assembly of focal adhesion contacts during cell migration [82,83], and as a buffer for protons to support the catalytic activity of the enzyme [84]. Recent studies have also suggested that this PG domain may serve as a proton antenna for monocarboxylate transporters, to facilitate proton-coupled lactate flux [85].

The regulation of CAIX expression has been linked not only to the hypoxic tumour microenvironment, but also to genetic mutations, and dysregulated growth factor signalling pathways. In hypoxia, CAIX expression is mainly upregulated by the stabilization of the hypoxia inducible transcription factors (HIF) and the activation of the HIF signalling pathway. The HIF signalling pathway has been extensively reviewed elsewhere [86,87]. Briefly, HIF-1 is a heterodimeric transcription factor that is composed of an O_2_-regulated HIF-1α subunit and a constitutively expressed HIF-β subunit [88]. In the presence of oxygen, the tumour suppressor von Hippel–Lindau (pVHL) protein targets HIF-1α for proteasomal degradation by means of proline hydroxylation using O_2_ and α-ketoglutarate as substrates [89]. Most notable amongst the HIF target genes are the vascular endothelial growth factor (*VEGF*) [90], carbonic anhydrase 9 (*CA9*) [68], and components of metabolic pathways such as lactate dehydrogenase-A (*LDHA*) [91] and glucose transporters (GLUT1) [92,93]. Of these, *CA9*, the gene encoding CAIX, is one of most responsive genes to hypoxia because the hypoxia response element (HRE) sequence of this gene is localized just upstream of the transcription initiation site [94]. Due to the tight regulation by HIF-1, CAIX is predominantly expressed in regions of chronic hypoxia within solid tumours [68].

Mutations and dysregulated signalling pathways in cancers have been mechanistically linked to the regulation of CAIX expression via hypoxia-independent activation of HIF. The most notable example would be the inactivation mutations of the von Hippel–Lindau tumour suppressor (*VHL*) gene which lead to the constitutive activation of HIF signalling and CAIX expression in clear cell renal cell cancers (ccRCCs) and the von Hippel–Lindau syndrome hemangioblastomas [95,96]. Besides this, HIF-1α expression and signalling are also regulated by major signal transduction pathways, including those that involve phosphatidylinositol 3-kinase (PI3K) [97] and extracellular signal-regulated kinase (ERK)/mitogen-activated protein kinase (MAPK) [98,99] and upstream tyrosine kinases such as SRC and RET [100,101]. Mutations in other tumour suppressor genes such as *PTEN*, *PML* and *TSC* activate HIF by promoting its translation [102,103,104]. Less common activators of HIF are mutations in the Krebs cycle enzymes, fumarate hydratase and succinate dehydrogenase [105,106,107], where the resulting increased levels of fumarate or succinate act as 2-oxoglutarate analogues to inhibit pVHL prolyl hydroxylation, thereby activating HIF signalling [108,109].

CAIX is expressed in a wide variety of solid tumours, including breast [110,111], colorectal [112], glioblastoma [113], lung [114], head and neck [115,116], and cervical cancers [117]; its expression is typically associated with poor disease prognosis [110,111,112,113,114,115,116,117]. The correlation between CAIX expression and poor prognosis can be mechanistically explained by CAIX protecting the cancer cell from intracellular acidosis, allowing rapid tumour growth, and creating extracellular acidosis, which promotes cancer cell migration and invasion. Both catalytic and non-catalytic CAIX-associated mechanisms allow cancer cells to progress along the invasion–metastasis cascade and thus to seed distant metastatic outgrowths [83,118,119,120].

CAIX expression has been shown to have an important role in the regulation and maintenance of cancer stem cells (CSCs), a small but distinct subpopulation of cancer cells within a solid tumour that are capable of regenerating the tumour. CSCs exhibit many of the properties of stem cells including clonogenicity, self-renewal, quiescence, and the ability to undergo epithelial-to-mesenchymal transition (EMT) [121,122]. In addition, CSCs have been shown to preferentially survive within hypoxic niches [123,124]. In the first study to report a link between CAIX and cancer stem cells, Lock et al. demonstrated that CAIX expression is required for the enrichment and function of breast CSCs in hypoxia, and that CAIX is required for mTOR signalling under hypoxia [125]. Intriguingly, CAIX expression was found to regulate the expression of ‘stemness’ genes such as Snail and Notch, and genes involved in the EMT; additionally, the inhibition of CAIX by small-molecule inhibitors depleted CSCs from tumours in vivo, reducing tumour growth and metastasis in preclinical models of cancer [125]. Ledaki et al. showed that subpopulations of cancer cells with hypoxia-inducible CAIX expression are enriched with cells that show high self-renewal capacity and express cancer stem cell markers. They also showed that the differential induction of CAIX under hypoxia is due to epigenetic regulation which induces differences in chromatin structure [126]. While these studies suggest that CAIX may be an important therapeutic target for selectively depleting CSCs, it remains unknown whether the effect of CAIX in CSCs is mediated through its role as a regulator of tumour pH_e_.

**Figure 1:**
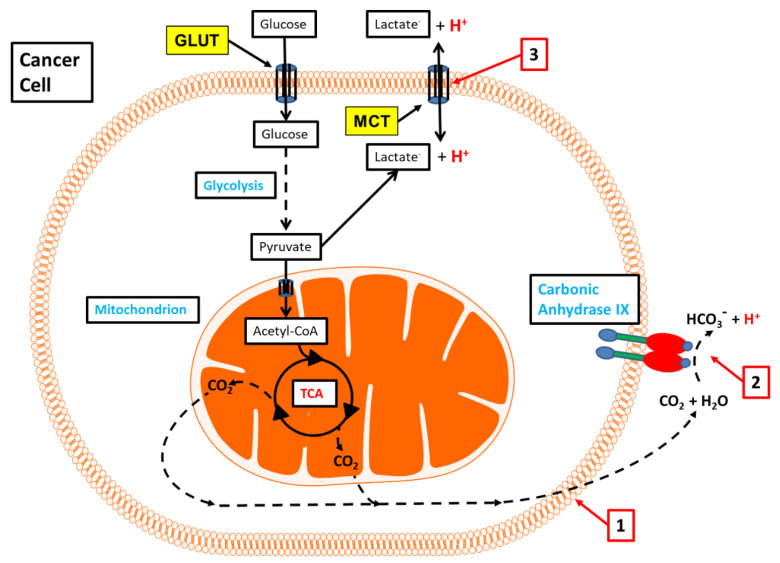
Carbonic anhydrase IX (CAIX) links tumour energy metabolism to its pH regulation. 1. The major by-product of oxidative energy metabolism, CO_2_ diffuses across the cell membrane lipid bilayer into the extracellular space, along its concentration gradient; 2. On the extracellular surface of the cell membrane, the exofacial catalytic domain of CAIX catalyses the hydration of CO_2_ to form H^+^ and HCO_3_^−^; 3. In parallel, lactate, the end-product of glycolysis, exits the cell through the monocarboxylate transporter at a rate that is influenced by the pH gradient across the cell membrane (pH_i_–pH_e_ gradient). GLUT, glucose transporter; MCT, monocarboxylate transporter.

CAIX catalyses the reversible hydration of CO_2_ at its exofacial site on the cell surface membrane, thereby placing it in a central position to regulate tumour pH_e_, and indirectly, pH_i_ (Figure 1). To study the effect of CAIX on pH_e_ and pH_i_, Swietach et al. employed confocal imaging using fluorescent pH dyes on 3D spheroid models of RT112 renal cancer cells [127] and HCT116 colorectal cancer cells [45], both of which overexpress CAIX. CAIX overexpression intensified the core-to-periphery pH_i_ and pH_e_ gradients of the spheroids, rendering the pH_i_ of cells at the core less acidic, but making the peripheral pH_e_ more acidic [45,127]. By the computational modelling of the data from their spheroid studies, they showed that CAIX maintains a steep outward-directed CO_2_ gradient across the plasma membrane, accelerating the CO_2_ excretion, and that this results in extracellular acidification and a more alkaline pH_i_ [45]. Mathematical modelling of this reaction in vivo suggested that the hydration of CO_2_ at the extracellular surface of the cell membrane also allows the parallel diffusion of CO_2_, HCO_3_^−^ and H^+^ across the interstitial space into the nearest blood vessel [45]. CAIX was predicted to exert an effect on the pH_i_ and pH_e_ in poorly perfused but metabolically active tissues, with the extracellular CO_2_/HCO_3_^−^ buffer system driven out of equilibrium [45]. The effect of CAIX on pH_e_ will depend on the type of acid released across the cell membrane, and the greatest acidifying effect would be observed if the cell metabolized glucose completely to CO_2_ [45]. In contrast, the greatest alkalinizing effect would occur if all the glucose was metabolized into lactic acid, without any neutralisation of the lactic acid by the HCO_3_^−^ to form lactate^-^ and H^+^ [45].

**Figure 2:**
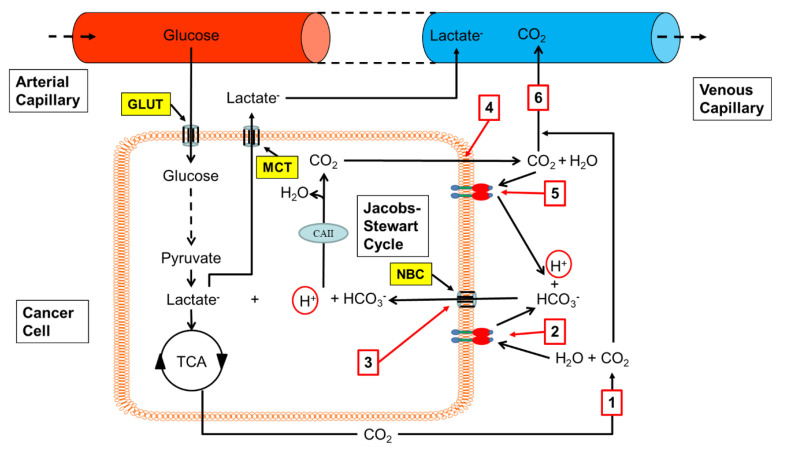
A Jacobs–Stewart Cycle helps hypoxic cancer cells extrude H^+^ produced by anaerobic glycolysis. 1. CO_2_ diffuses from the cancer cell into the extracellular space; 2. CAIX on the extracellular surface of the hypoxic cancer cell catalyses the hydration of CO_2_ to form H^+^ and HCO_3_^−^; 3. The HCO_3_^−^ enters the hypoxic cancer cell via the Na^+^/HCO_3_^−^ cotransporter and binds H^+^ from lactic acid, catalysed by CAII, thus forming CO_2_; 4. The CO_2_ diffuses from the hypoxic cancer cell into the extracellular space, along its concentration gradient; 5. On the extracellular surface of the hypoxic cancer cell, CAIX catalyses the hydration of the CO_2_ to form H^+^ and HCO_3_^−^. The HCO_3_^−^ provides further substrate to repeat stage 3 of the cycle, and an equivalent amount of H^+^ that was originally inside the hypoxic cancer cell (ringed in red) is now in the extracellular fluid; 6. Accumulating CO_2_ diffuses to a capillary. GLUT, glucose transporter; MCT, monocarboxylate transporter; NBC, Na^+^/HCO_3_^−^ cotransporter.

Hypoxic cancer cells display CAIX on their extracellular membrane but also have intracellular carbonic anhydrases such as CAII. This would allow them to extrude the H^+^ that is formed by the dissociation of the lactic acid product of anaerobic glycolysis, by using a Jacobs–Stewart Cycle (Figure 2) [128]. Bicarbonate, formed by the mechanism shown in Figure 1, is transported by the Na^+^/HCO_3_^−^ cotransporter into the hypoxic cell, where, under catalysis by CAII, it binds to H^+^, forming CO_2_ and H_2_O. The CO_2_ then diffuses through the cell membrane into the extracellular space where CAIX on the extracellular surface catalyses its hydration to HCO_3_^−^ and H^+^; thus, an equivalent amount of H^+^ that was originally in the hypoxic cell is now in the extracellular fluid. Excess CO_2_ from the cancer tissue will diffuse to host capillaries.

## 6. Discovery of the Tumour Extracellular pH-Stat Mechanism

Stubbs et al. previously proposed that the acidic tumour pH_e_ reflects more than just the high acid output of the cancer cells, and postulated the existence of an extracellular pH homeostatic mechanism, a pH_e_-stat, that was subverted by the cancer cell to set the pH_e_ at a constant level and curtail excessive pH loading [129]. While an acidic tumour pH_e_ promotes tumour growth and invasion, the pH_e_ cannot be allowed to fall too low, as over-acidification would cause cell necrosis. Hence, cancer cells must utilise this pH_e_-stat to maintain a microenvironment that is hostile to the surrounding normal tissues, yet favourable for their own survival.

If pH_e_ was indeed under homeostatic control, then it should not correlate with pH_i_, whereas in a model of H^+^ transport, in which the rate of the H^+^ efflux from the cells depends only on the proton concentration in the interstitial space, there should be a tendency for pH_i_ and pH_e_ to be correlated. Using ^31^P MRS and the extracellular pH probe 3-APP, Stubbs et al. measured pH_i_ and pH_e_ in a range of rodent tumour models (RIF-1 fibrosarcoma, H9681a hepatoma and GH3 prolactinoma), and found no correlation between the pH_i_ and pH_e_ values [129]. These findings supported the hypothesis that the acidic tumour pH_e_ is not simply a function of tumour metabolism *per se*, but is maintained by a homeostatic mechanism—a cancer pH_e_-stat.

Several in vitro studies have suggested that the biochemical and biophysical properties of CAIX make it suited to the role of pHe-stat. In 2009, Alterio et al. published the first x-ray crystal structure of the catalytic domain of CAIX and showed that the presence of the proteoglycan chain at the border of the active site of the enzyme lowered its pK_a_ from 7.01 to 6.49, ensuring that the enzyme had better catalytic efficiency at the acidic pH values at which it operated [130]. Using ^18^O exchange between CO_2_ and H_2_O to measure the exofacial CAIX activity in MDA-MB-231 breast cancer cells by membrane inlet mass spectrometry, Li et al. reported the first detailed measurement of the catalytic properties of the enzyme in its native membrane environment [131]. They found that the rate constant for the catalytic hydration reaction was faster than for the dehydration at the physiological pH of 7.4 [131]. At pH 6.8, the rate constants for hydration and dehydration were essentially equal, and at pH values lower than 6.8, the dehydration reaction was faster [131]. Mahon et al. reported that the catalytic domain of CAIX exhibits biochemical and biophysical properties that create low pH stability and activity [132]. Taken together, these findings suggest that the primary role of tumour CAIX is to utilize the interconversion of CO_2_ and HCO_3_^−^ in order to stabilize the pH_e_ around 6.8, thus maintaining an acidic pH_e_ set-point of cancer cells in response to the proton load from cellular metabolism. Intriguingly, the measurement of CAIX activity in vitro in HCT116 cells showed that CA activity was markedly reduced by decreasing the pH over the pH range found in tumours, with a pK of 6.84 and a Hill cooperativity coefficient of 2. This Hill coefficient would tend to sharpen the change in enzyme activity with pH_e_, suggesting that CAIX may have been adapted to provide a pH_e_-stat mechanism whereby excess acid self-limits the build-up of extracellular acid [80].

In parallel, several investigators have sought to study the effect of CAIX expression on tumour pH_e_ in xenograft models, using various magnetic resonance-based pH_e_ measurement techniques. Chen et al. employed the chemical exchange saturation transfer (CEST)-based MRI pH_e_ measurement technique, acidoCEST MRI, to measure tumour pH_e_ in a range of lymphoma xenograft tumour models (Raji, Ramos and Granta 519 cell lines) with different levels of CAIX expression [133]. The average tumour pH_e_ ranged between 6.78 and 6.86, but there was no statistically significant difference between the mean pH_e_ values of the different models. They derived a xenograft acidity score from the percentage and magnitude of the acidity, and found a correlation between that score and the level of CAIX expression in the tumour models, measured by histopathological staining [133]. Despite the positive correlation, it was not possible to draw any conclusion about causality between the levels of CAIX expression and the xenograft acidity score.

Using hyperpolarized ^13^C magnetic resonance spectroscopy with [^13^C] bicarbonate (H^13^CO_3_^−^), Gallagher et al. measured the pH_e_ of two colorectal tumour xenograft models expressing different levels of CAIX [27]. However, while they observed a lowering of pH_e_ in the CAIX-overexpressing tumours, the measured tumour pHe values were alkaline, likely an overestimation error due to the incomplete equilibration of the hyperpolarized ^13^C label between the H^13^CO_3_^−^ and ^13^CO_2_ pools on the timescale of the ^13^C MRS measurements. This problem arises because the accurate measurement of pH_e_ using the hyperpolarized ^13^C bicarbonate technique is dependent on the tissue having high levels of carbonic anhydrase activity [27]. Using ^13^C MRS magnetization transfer to measure the rate of exchange of hyperpolarized ^13^C label between bicarbonate and CO_2_, Gallagher et al. were able to measure the enzymatic activity of carbonic anhydrase in their tumour models and observed that, paradoxically, the tumours overexpressing CAIX showed a lower enzyme activity compared to the controls [27]. The administration of bicarbonate in the drinking water of mice elevated tumour pH_e_ and restored enzyme activity to control levels, suggesting that the enzymatic activity of CAIX exhibits pH dependence and can be inhibited at low pH levels, akin to a negative feedback mechanism [27]. This observation suggests the interesting proposition that CAIX expression may be increased in hypoxia to compensate for the decreased specific activity of the enzyme at the lower pH_e_.

To study the regulatory effect of CAIX on the tumour pH_e_, Lee et al. employed a high-resolution in vivo pH-imaging technique that was independent of the enzymatic kinetics of CAIX. Using ^1^H MRSI with an extracellular pH ^1^H MRS probe, ISUCA, they performed high-resolution spatial mapping of pH_e_ in colorectal tumour xenograft models expressing different levels of CAIX, and observed that CAIX-expressing tumours had a more acidic pH_e_ than the control tumours [134]. Importantly, the expression of CAIX imposed an upper limit of tumour pH_e_ at 6.93, consistent with the hypothesis that CAIX acts as a pH-stat to maintain the pH_e_ at a more acidic level than the typical pH_e_ of 7.35–7.45 [134]. Intriguingly, there was no difference in pH_i_ values between the CAIX-expressing and non-CAIX expressing tumours, as measured by ^31^P MRS, suggesting that CAIX acidifies only the tumour pH_e_ and not pH_i_ [101]. This latter observation was concordant with the earlier proposal of Stubbs et al. [129] that if pH_e_ was indeed under homeostatic control, then it should not correlate with pH_i_. Additionally, these CAIX-expressing tumours were found to have a higher level of lactate compared to the control tumours [134]. Calculations of the ratio of lactate on either side of the membrane from the mean pH_i_ and pH_e_ values of these tumours revealed that CAIX-expressing tumours had markedly higher intracellular lactate than non-CAIX expressing control tumours [134]. Collectively, these findings suggest that CAIX acts as a pH-stat that maintains an acidic tumour pH_e_.

## 7. Implications of an Extracellular pH-Stat for Cancer Progression

It is now well established that the acidic tumour pH_e_ phenotype is associated with aggressive behaviour of solid tumours. Thomlinson and Gray first reported that human tumours grew as cords around blood vessels, and that as a tumour grows and outstrips its blood supply, its central region becomes hypoxic, although the cells remain within the diffusion distance of glucose [135]. Hypoxia upregulates glycolysis through the activation of hypoxia-inducible factor-1 (HIF-1) which induces the expression of glycolytic enzymes (e.g., lactate dehydrogenase A) [136], proton-exporting active transporters, (e.g., monocarboxylate transporter 4 (MCT4) [137]), and carbonic anhydrase IX (CAIX) [68].

These adaptations, which enable cancer cells to survive hypoxia by maintaining their intracellular pH within the physiological range and thereby creating an acidic pH_e_, have also been found to promote their invasion into normal host tissue and metastasis into other organs. Setting pH_e_ to a constant acidic condition may therefore be an evolutionary strategy used by cancer cells to create an environment that fosters tumour growth and invasion in response to microenvironmental selection forces. One could even argue that the characteristically hypoxic tumour microenvironment and the chaotic blood supply that gives rise to it are both evolutionarily selected features that are driven by the aggressive phenotype conferred on cancer cells by extracellular acidity.

Gatenby, Gillies and colleagues have shown that an acidic pH_e_ promotes tumour local invasion and metastasis, and that cancer cells are able to modulate the set point of tumour pH_e_ in response to microenvironmental selection pressures as an evolutionary strategy favouring invasion [21,138]. At the cellular level, migrating cancer cells have proton-exporting machinery, such as sodium–hydrogen exchangers (NHE1) or sodium-bicarbonate cotransporters (NBCn1), coupled to CAIX at the front of their invadopodia, resulting in a more acidic pH_e_ at the leading edge of the migrating cell [83,119] which activates proteases, e.g., lysosomal cathepsins, that then degrade the host’s extracellular matrix [139,140]. Gatenby et al. initially reported in silico mathematical simulations predicting that tumours acidified the extracellular space of normal tissue around the tumour edge [141], and confirmed these predictions via the intravital imaging of PC3N and MCF7 tumours, grown in dorsal window chambers [21]. They measured the peritumoural H^+^ flow using vectors generated from pH_e_ distribution around the tumours, and found a general flow of tumour-derived acid from the core to the periphery, and then into the surrounding normal tissue [21]. In another study using dorsal window chamber tumour models and the confocal imaging of pH_e_ with a fluorescent pH indicator, SNARF-1 Free Acid, they observed that the acidic pH_e_ of peritumoural tissues was coincident with the location of subsequent tumour invasion, confirming a specific prediction of the acid-mediated invasion hypothesis [22]. The treatment of the animals with sodium bicarbonate reduced the pH gradient and reduced the local invasion in these tumour models [22]. Using a theoretical framework from evolutionary dynamics, Gatenby and Gillies proposed that the metabolic properties of cancer cells and their consequent interstitial acidification provided an evolutionary advantage that promotes tumour cell proliferation at the expense of the surrounding normal cells [142].

Further support for the acid-mediated invasion hypothesis has come from histopathological data showing cells at the invading edge expressing significantly more CAIX than those in the core of the tumour. Using quantitative image analysis of histologic specimens from patients with breast cancers, Lloyd et al. found that the spatial distribution of CAIX was consistently higher in the invasive tumour edge compared to the centre in all samples, which matched the predictions of evolutionary game theory models [143]. A key assumption of their model was that CAIX was able to set the pH_e_ at 6.8 and that it served as a biomarker of regional tumour acidity. Thus, cancer cells are predicted to evolve towards an evolutionarily stable state, dictated by their adaptations to changes in environmental conditions such as blood flow and hypoxia [143]. Collectively, these studies suggest that the acidic pH_e_ confers a selective advantage during the somatic evolution of cancers by facilitating the invasion of cancer cells into surrounding normal tissues.

## 8. CAIX as a Therapeutic Target in Oncology

CAIX is a promising therapeutic target in oncology. In vivo studies have shown that the genetic ablation of CAIX reduced the growth rate of colon and breast cancer tumour xenografts. Using a tetracycline-inducible system for RNA-interference, Chiche et al. reported that silencing the *ca9* gene reduced colorectal LS174Tr xenograft tumour volume by up to 40%, and that this also resulted in a compensatory upregulation of *ca12* mRNA [78]. Consequently, the greatest reduction in tumour volume was achieved through silencing both *ca9* and *ca12* genes. In cultured cells challenged with a CO_2_ load, both CAIX and CAXII contribute to extracellular acidification and the maintenance of a more alkaline resting pH_i_ [78]. In a 4T1 murine metastatic breast cancer model and an MDA-MB-231 human breast xenograft model, Luo et al. reported that shRNA-mediated the silencing of CAIX-attenuated tumour growth; it also inhibited spontaneous lung metastasis formation in the 4T1 model [78]. The effect on tumour growth and metastasis formation was recapitulated through the use of CAIX-specific small molecule inhibitors, confirming the efficacy of CAIX as a targetable biomarker for cancer therapeutics [78].

Another avenue for the use of CAIX inhibition may be as a combination therapy with antiangiogenic drugs; this could produce additive results, since immunohistochemical data show that CAIX is part of a resistance mechanism that enables tumours to adapt to the increased hypoxia induced by bevacizumab treatment [80]. McIntyre et al. reported that the shRNA knockdown of CAIX in colon cancer HCT116 and U87 glioblastoma xenografts enhanced the effect of antiangiogenic therapy with bevacizumab, resulting in a reduced tumour growth rate in vivo [80]. They also used acetazolamide to inhibit the enzymatic activity of CAIX, which recapitulated the effects of a combination of CAIX knockdown and bevacizumab. Like Chiche et al., they found that CAIX knockdown induced the expression of CAXII in 3D spheroids, but not in 2D cell culture [80]. In spheroid culture and tumour xenografts, CAIX expression was found to increase the growth rate and the expression of Ki-67, a marker of proliferation, while at the same time promoting necrosis and apoptosis [80].

## 9. CAIX—Clinical Translation

There is now convincing evidence that CAIX can serve as a biomarker and/or therapeutic target in various tumour types. To this end, a distinction can be drawn between the tumours that express CAIX as a consequence of inactivating mutations of the von Hippel–Lindau (pVHL) tumour suppressor protein, and tumours in which CAIX is present due to microenvironmental hypoxia. In the former group, exemplified by clear cell renal cell carcinoma (ccRCC), more than 90% of tumours with inactivating mutations of pVHL have a constitutive stabilization of HIF and a high percentage of tumour cells expressing CAIX [144,145]. In more advanced ccRCC, CAIX expression decreases due to a switch from the HIF-1 to the HIF-2 isoform, which explains why the low expression of CAIX (defined as staining fewer than 85% of cells) in such tumours is a marker of poor prognosis [146].

In many other tumour types, such as head and neck, breast, colorectal, brain and lung tumours, CAIX is expressed in areas that are hypoxic and/or acidic, and its expression usually increases with increasing tumour stage and grade [68]. Immunohistochemical staining of CAIX is mostly found at the plasma membrane, and while cytoplasmic and/or nuclear staining are also occasionally seen, their biological significance is unclear. In addition, CAIX expression is associated with various other prognostic variables including c-ErbB2/Her2 [147,148], EGFR [114,149], MMP-9 [149], CD44 [150] and Ki-67 [151]. CAIX can also be detected in tumour stroma, which is associated with poor prognosis [152]. As a consequence of ectodomain shedding and release in exosomes, CAIX can be detected in the body fluids of cancer patients, and this can be exploited for non-invasive screening or monitoring tumour response to therapy [153,154].

Patients with tumours highly expressing CAIX are at a higher risk of disease progression and metastasis, independent of the tumour type or site. In a meta-analysis of studies between 2001–2015 involving more than 24,000 patients with non-RCC tumours, there was a strongly significant association between immunohistochemical CAIX staining and several different endpoints, including overall survival, disease-free survival, locoregional control, disease-specific survival, metastasis-free survival, and progression-free survival [155]. Besides this, numerous studies have reported the correlation between CAIX expression and resistance to chemotherapy [156], radiotherapy [115,157] and immunotherapies against PD-1 [158]. These findings provide a rationale for developing prognostic biomarkers and therapeutics based on CAIX expression.

Inhibitors of carbonic anhydrase activity as emerging anticancer therapeutics have been reviewed elsewhere [159]. In general, therapeutic strategies targeting CAIX involve one of two approaches. The first is based on the role of CAIX in pH regulation and uses compounds that inhibit its enzymatic activity through binding at or near its active site. The efficacy of this approach has been demonstrated by several preclinical studies in murine models of cancer [78,79,80]. Recently, SLC-0111, a small molecule inhibitor of CAIX, successfully completed Phase I clinical trials for the treatment of advanced, metastatic solid tumours [160]. Efforts are also underway to recruit patients for a Phase 1b trial of SLC-0111 in combination with gemcitabine in metastatic pancreatic cancer [161]. However, the clinical use of single agent CAIX inhibitors is likely to be hindered by the cancer cell, inducing compensatory mechanisms such as the upregulation of CAXII expression, or other acid-extruding mechanisms such as NHE1, resulting in an unsatisfactory therapeutic effect. Therefore, a more prudent approach would be to explore the use of anti-CAIX therapy in conjunction with other established anti-cancer drugs. One example of this concept would be to use anti-CAIX therapy in patients undergoing anti-VEGF therapy, since CAIX expression is upregulated as a compensatory mechanism in tumours experiencing hypoxia as a result of anti-angiogenesis [80,162]. Besides this, CAIX inhibition can also be used in conjunction with radiotherapy, which has been demonstrated by the use of DH348, a nitroimidazole-based anti-CAIX inhibitor that not only reduced tumour growth in mice but also sensitized the tumours to radiotherapy [163].

The second strategy targets CAIX by immunotherapy and exploits the tumour-associated expression pattern of CAIX. The use of monoclonal antibodies against CAIX ensures high specificity and selectivity that are superior to the effects of chemical inhibitors, and the quick cell-killing effect of antibody-dependent cellular cytotoxicity (ADCC) circumvents the development of compensatory mechanisms. Most of the studies using this strategy have been undertaken in animal models of RCC and in patients with non-metastatic RCC, using the chimeric human-mouse monoclonal antibody G250 (RENCAREX/GIRENTUXIMAB), which is a safe, well tolerated antibody that was found to prolong disease-free survival by up to 22 months in a subgroup of ccRCC patients with high tumour CAIX expression [164].

In non-RCC tumours, CAIX expression is less frequent and more heterogeneous, thus it may not be such an attractive target for immunotherapy. However, the association between CAIX and metastasis makes it a plausible target as an adjuvant for metastasis-targeted immunotherapy. Since tumour cells residing in hypoxic and acidic regions are associated with therapy resistance, it is conceivable that these cells will express CAIX and that they will survive chemotherapy and/or radiotherapy and seed metastatic outgrowths. While targeting CAIX in such cells is certainly an intriguing prospect, there are only a few reported studies on the detection of CAIX in metastatic lesions [165,166,167], therefore more evidence is needed to support this hypothesis.

Our understanding of the function of CAIX in tumour pH_e_ regulation has highlighted the importance of mitochondrial energy metabolism in cancer. In parallel, several studies have suggested that mitochondrial metabolism plays an important role in the development of drug resistance in cancer [168,169]. It has been argued that resistant cancer cells are more dependent on mitochondrial oxidative metabolism than glycolysis. In many haematological and solid tumours, treatment resistance is associated with a shift towards an oxidative phosphorylation-dependent energetic status [60,170,171,172]. Resistant cells display higher levels of reactive oxygen species (ROS) and increased mitochondrial mass, oxygen consumption, ATP production and fatty acid synthesis [173,174,175]. This metabolic adaptation has been hypothesized as a strategy to meet the increased energetic demands of DNA repair, drug efflux and detoxification. Furthermore, the inhibition of oxidative phosphorylation has been shown to abrogate resistance to EGFR inhibitors in EGFR-driven lung cancers [176], docetaxel in prostate cancer [177], MAPK inhibitors in melanoma [178] and 5-fluorouracil (5FU) in colon cancers [179]. Therefore, combining conventional chemotherapies with mitochondrial inhibitors (e.g., metformin, tigecycline) represents a promising strategy [180]. In the context of CAIX and tumour pH_e_ regulation, it is tempting to speculate that the inhibition of mitochondrial respiration may reduce the acidifying effect of CAIX on pH_e_.

## 10. Conclusions

Our understanding of tumour pH and its regulation has come a long way, since the landmark discoveries of cancer cell metabolism by Otto Warburg, and the first in vivo measurements of tumour intracellular and extracellular pH in solid tumours. The progress in recent decades in the fields of cancer modelling, molecular biology, genetics, and in vivo imaging techniques has provided us with a molecular-level understanding of acid-base homeostasis in cancer. Since its discovery in the early 1990s, CAIX has emerged as an important pH regulator in solid tumours, with recent evidence demonstrating that it functions as a pH_e_-stat. With increasing knowledge about the contribution of acidic pH_e_ to all steps of cancer development and progression, it is now evident that CAIX provides a selective advantage to cancer cells by allowing them to create a hostile acidic environment in which they can survive and thrive, but in which host cells cannot, and to acquire an invasive and metastatic phenotype. The fact that CAIX-expressing cancer cells generally represent the most aggressive fraction of solid tumours indicates a fertile avenue for clinical translation in terms of diagnosis, prognosis, and therapy. It is unlikely, however, that targeting CAIX on its own will be sufficient to produce a meaningful clinical benefit because of the phenotypic plasticity and redundancies in cellular pH regulatory mechanisms. Probably, therefore, the greatest benefit of agents targeting CAIX would be realized by using them in combination with other therapeutic agents in an adjuvant or neoadjuvant setting.

## Figures and Tables

**Table 1:** Summary of the non-invasive techniques for measuring tissue pH.

Imaging Technique	Description
Fluorescence imaging	Various fluorescent dyes and proteins exhibit a wavelength shift upon protonation. Ratiometric measurement of the fluorescent output at each wavelength allows the calculation of pH.
	Advantage: very high spatial resolution (<1 µm) enables the measurement of subcellular pH gradients
	Disadvantage: low penetration of the tissue limits its use in whole animal and clinical imaging.
	The use of SNARF-1 fluorescent dye on tumours grown in dorsal window chamber models has enabled the in vivo imaging of tumour pH [21,22].
Magnetic resonance spectroscopy	MRS measures the pH-dependent chemical shift of MRS-visible nuclei such as ^1^H, ^31^P, and ^19^F in endogenous or exogenous compounds.
	In compounds with pK_a_ close to the physiological range, this property can be exploited to measure pH by comparing the chemical shift of the pH-sensitive peak with a previously constructed titration curve of chemical shift vs. pH.
	^31^P MRS can be used to measure pH_i_ from the chemical shift of endogenous inorganic phosphate, while pH_e_ can be measured with the use of 3-aminopropylphosphonate (3-APP). However, because of the low sensitivity of the ^31^P nucleus, this technique has poor spatial resolution, and cannot be used to generate pH_e_ maps [7,23,24].
	^19^F MRS can be used to measure pH_e_ with ZK-150471 (3-[*N*-(4-fluoro-2-trifluoromethylphenyl)-sulfamoyl]-propionic acid) and fluorinated vitamin B6 derivatives (6-fluoropyriodoxol & 6-fluoropyridoxamine) [25].
	^1^H magnetic resonance spectroscopic imaging (MRSI) of the imidazole-containing compounds, (±)2-(imidazol-1-yl)3-ethoxycarbonylpropionic acid (IEPA) [8] and (±)2-(imidazol-1-yl) succinic acid (ISUCA) [9] can be used to generate pH_e_ maps with a spatial resolution of 1–2 mm. However, the poor temporal resolution of 10–30 min precludes measurement of pH changes.
Hyperpolarised (HP) ^13^C MR imaging	Dissolution dynamic nuclear polarization (d-DNP) results in a substantial increase in the magnetic resonance (MR) signal [26], allowing measurements of spatial and temporal changes in pH_e_ from exogenously administered hyperpolarized molecules.
	Tumour pH_e_ can be measured with hyperpolarized H^13^CO_3_^−^ using the ratio of the peak intensities of H^13^CO_3_^−^and ^13^CO_2_ [27].
	However, this technique is dependent on carbonic anhydrase activity, and in tissues with low carbonic anhydrase activity, the measured pH will tend to be overestimated [28].
	pH_i_ can be quantified from HP ^13^CO_2_ produced from [1-^13^C] pyruvate in organs with a high pyruvate dehydrogenase flux, e.g., the heart [29]. The short T_1_ of [^13^C] bicarbonate limits spatial resolution [27]. The current resolution of 2–10 mm is coarser than that of some other MR imaging approaches.
	Other ^13^C-labelled compounds have been shown to be able to measure in vivo pH_e_ using HP ^13^C MRS: *N*-(2-acetamido)-2-aminoethanesulfonic acid (ACES) [30], diethylmalonic acid (DEMA) [31], zymonic acid [32], and amino acid derivatives [33].
Chemical exchange saturation transfer (CEST)	CEST measures pH_e_ from the relative rates of protonation and deprotonation of amide functional groups on either endogenous molecules or administered contrast agents. It has the advantage of high spatial resolution (0.1–2 mm) but has low sensitivity.
	AcidoCEST is a CEST-based method of measuring pH_e_ based on exogenous contrast agent administration [34].
	Iopidamol, a United States Drug and Food Agency (FDA)-approved contrast agent, has been used with this approach to measure pH_e_ in metastatic ovarian cancer tumour models [35].
MR relaxometry	pH is measured from paramagnetic contrast agents with a pH-dependent change in T_1_ relaxation time. It has the advantage of high signal-to-noise ratio and spatial resolution (0.2–2 mm). However, to accurately measure the pH, this technique requires the administration of a second pH-insensitive agent to correct the concentration of the pH-sensitive agent.
	An example of the agent is GdDOTA-4AmP^5−^, a pH-dependent chelate which has been used together with a pH-independent analogue, GdDOTP^5−^, to generate high spatial resolution maps of tissue pH_e_ in a rat glioma model [36].
PET/SPECT imaging	In vivo pH_e_ imaging using positron emission tomography (PET) has been achieved using ^11^C-dimethyloxazolidinedione (DMO) [37], ^11^CO_2_ [38], and ^123^I-labeled derivatives of malonic acid [39].
	pH (low) insertion peptides (pHLIP) is a class of acid-targeting peptides that are activated to insert across the membrane of cancer cells by the acidity at the cell surface. These peptides can be conjugated with ^64^Cu to target the radioisotope to regions where the pH_e_ is below 7.0 [40].
	Caged derivatives of ^18^F-fluorodeoxyglucose (FDG) can be localized to tumours by the release of the caging group in low pH_e_, followed by uptake via glucose transporters [41].
	PET-based techniques are readily implemented with good spatial resolution (1–2 mm). A limitation of this approach is that it cannot measure absolute pH, and only indicates regions below a certain pH_e_ threshold. pH values can be estimated from arterial blood sampling and model fitting, but these values have not been correlated with microelectrode measurements.
